# Medical and Philosophical Intelligence

**Published:** 1819-10

**Authors:** 


### Medical and Philosophical Intelligence.

WE feel much gratification in announcing to our readers, that a new edition of the *Memoires et Prix de l'Académie Royale de Chirurgie* of Paris, is now in the course of publication.‡ The value of this collection of dissertations is too generally known and well substantiated, to make any particular comments necessary to show of what importance it is that a new edition should be produced, since the old quarto one has become so scarce and expensive. The editors have contributed much to the value of the present by the addition of notes, pointing out the authors who have since written on the same subjects, and adducing many corollaries possessing considerable interest, which will be found advantageous, especially to students. To this class of our readers we may render some useful advice, in saying that it should form an essential part of their library.

ENTOMOLOGY, besides its interest as a branch of natural history, has many peculiar claims on the attention of the true physiologist,—that is, one who extends his views to the whole of animated nature; and it is also of importance in its relations to various diseases of the human body. A work recently published by Mr. SAMOUELLE§ will much

‡ It will form eight volumes in 8vo.; and is published by *Menard* and *Desenne*, of Paris, and may be obtained in London, of *Mr. Souter*.

§ *The Entomologist's Compendium*, &c. &c. By GEORGE SAMOUELLE, Associate of the Linnean Society of London. 8vo. pp. 496. Price, with coloured figures, 1l. 18s.; plain, 1l. Boys, Ludgate-hill, London; 1819.

facilitate the study of this subject; giving a concise, but accurate and perspicuous, description of the whole of the known genera and species of those of our country, with some useful general observations on their structure, and the mode of collecting and preserving them. We have not often seen the results of so much labour collected into so small a volume. It does great credit to the author's industry and acquirements as a naturalist; and, as far as our approbation may extend, we should feel pleasure in contributing to favour the reward due to his useful exertions.

---

The first number of the American Journal of Science, edited by Dr. Silliman, contains some observations on the efficacy of *respiration of oxygen gas*, which appear to be interesting. We shall give them to our readers, with the judicious sentiments expressed by the editor.

"It is not extraordinary, when oxygen gas was first discovered, and found to be the principle of life to the whole animal creation, that extravagant expectations should have been formed as to its medicinal application. Disappointment followed of course, and naturally led to a neglect of the subject; and, in fact, for some years, pneumatic medicine has gone into discredit, and public opinion has vibrated to the extreme of incredulity. Partaking in a degree of this feeling, we listened with some reluctance to a very pressing application on this subject during the last summer. A young lady, apparently in the last stages of decline, and supposed to be affected with hydrothorax, was pronounced beyond the reach of ordinary medical aid. As she was in a remote town in Connecticut, where no facilities existed towards the attainment of the object, we felt no confidence that, even if oxygen gas were possessed of any efficacy in such cases, it could actually be applied in this case in such a manner as to do any good. Yielding, however, to the anxious wishes of friends, we furnished drawings for such an apparatus as might be presumed attainable. The gas was directed to be obtained from nitrate of potash, not because it was the best process, but because the substance could be obtained in the place, and because a common fire would serve for its extrication. The gas obtained had, of course, a variable mixture of nitrogen, and probably on an average might not be purer than nearly the reversed proportion of the atmosphere: that is, 70 to 80 per cent. of oxygen to 20 or 30 of nitrogen; and it is worthy of observation, whether this circumstance might not have influenced the result.

"From the first, the difficulty of breathing, and other oppressive affections, were relieved; the young lady grew rapidly better, and in a few weeks entirely recovered her health. A respectable physician, conversant with the case, states, 'that the inhaling of the oxygen gas relieved the difficulty of breathing, increased the operation of diuretics, and has effected her cure. Whether her disease was hydrothorax, or an anasarcous affection of the lungs, is a matter, I believe, not settled.'

"This interesting series of experiments (on the respiration of oxygen gas) ought to be resumed, not with the spirit of quackery or of extravagant expectation, but with the sobriety of philosophical research; and it is more than probable that the nitrous oxide, which is now little more than a subject of merriment and wonder, if properly diluted and discreetly applied, would be productive of valuable effects."

In the Monthly Gazette of Health for the present month, there is the following passage (page 271): "Small-pox continues to prevail throughout the island, and the contagion not being resisted by subjects who have gone through vaccination to the perfect satisfaction of medical men who communicated the disease, instances of failures have in every county so multiplied, that they no longer excite attention. *The demand for cow-pox matter at the National Vaccine Institution has in consequence nearly ceased.*"

Lest these positive assertions should be credited by any one, the Board of the National Vaccine Establishment have authorized us to state, that the number of persons vaccinated during the last eight months, from January to September, at their ordinary stations alone, amount to 5,996; and the number of charges of vaccine lymph, which were distributed to the public during the same period, were 34,597; which is a considerable augmentation, instead of a decrease, of the business of the establishment.

We have the same authority for assuring the profession, that, notwithstanding the strange opposition which is still persevered in, the practice of vaccination steadily gains ground throughout the British dominions.

The following cases of *aberration of the menstrual evacuation in women*, have been published in the Bulletin de la Societé du Département de l'Eure, and seem to be very important in their application to the opinion respecting the nature of the uterine flux that is becoming most commonly adopted. They are related by Dr. de Reynal.

A girl, aged 15, experienced suddenly, without any precursory signs, her first menstruation, which was abundant, but suppressed after a few hours by a fright. No consequent accident immediately happened; but, at the end of three weeks, a tumor appeared on the right side of the chest, in the situation of the middle of the fifth and sixth ribs. It suppurated and burst: on the following day an oozing of blood took place from the cavity of the abscess, and continued four or five days. From this time a bleeding took place from the same part every month, and the menses never re-appeared. At the age of 23, it was first suspended during the time of pregnancy, which then supervened. The woman has constantly enjoyed good health.

A woman, who had never menstruated regularly, had, at the age of 16, a slight hæmorrhage from the nose, which continued to incommode her for three days, and then disappeared suddenly as it commenced. From that time the bleeding from the nose has been constantly renewed every month, except during the periods of pregnancy; and it did not cease till she was 42 years of age. The woman had been fourteen times pregnant, and enjoyed the most perfect health

It is stated, in the Nouveau Journal de Medicine (Mai 1819), that *small-pox* has been lately epidemic, and very fatal, at Martinique. The disease was transmitted from the coast of Africa. It did not attack persons who had been vaccinated by medical practitioners, and was particularly prevalent amongst the negroes.

*Yellow fever* had also reigned there during the whole of the *cold season.* It is remarked, that one person, who had resided many years in the Antillas, and become apparently well-seasoned, having left the island he ordinarily inhabited, died of yellow-fever at Martinique; which, as well as the former observation, is contrary to some opinions entertained respecting the nature of that disease.

---

M. Thenard has recently announced to the Royal Academy of Sciences of Paris, a chemical discovery which may probably add an useful article to the *materia medica.* On making some attempts to charge water with a proportion of oxygen superior to that which it contains in the ordinary state, he has obtained a liquid which holds in combination 0·88, by weight, of oxygen, which is enormous. This liquid is colourless, almost inodorous: its taste is styptic, and analogous to that of tartar emetic; its density is 1·412. Its action on the skin is very remarkable; it whitens it, causes very sharp pricking sensations, and produces vesications in the same manner as epispastics. When placed in contact with different metals,—as gold, silver, osmium, platinum, iridium, rhodium, palladium, and their oxides: it gives rise instantly to a powerful detonation, with disengagement of heat, and an emission of light, perceptible in an obscured situation. We have not yet received information of the method of forming this compound; but, when we do, it shall be imparted to our readers.

---

The following gentlemen have obtained the degree of Doctor in Medicine, from the university of Glasgow, within the last twelve months:

Mr. Wm. Hume, from Scotland; Mr. John Banks, from England; Mr. Edward Talbot Kelly, from Ireland; Mr. Andrew Ferguson, from Scotland; Mr. David Macnab, from Scotland; Mr. William Jones, from England; Mr. John Smyth, from Ireland; and Mr. Hans Harper, from Ireland.

---

*Prize Question proposed by the Society of Practical Medicine at Paris.*—Persuaded that the present state of science permits us to view in a more favourable light than formerly a point of doctrine which still leaves some uncertainty in therapeutics, the Society of Practical Medicine of Paris has thought proper to call the attention of medical practitioners to the affections of which traces are found in the abdominal viscera, after putrid and ataxic (nervous) fevers. The Society desires that those who engage in the investigation of this subject, will endeavour to determine the character of those affections, and the relations they have with what are termed idiopathic fevers; and especially that they will not deviate from the rational medicine founded by Hippocrates, bequeathed by that divine physician to his true successors, and preserved by practitioners worthy of that title. The Society consequently proposes, for the subject of a prize, consisting in a gold medal of the value of two hundred francs, which will be awarded at the last sitting in the year 1820, the following question:

*Are the affections of which traces are found in the abdominal viscera after putrid or adynamic, and ataxic, fevers; the effects, the cause, or a complication, of those fevers?*

The memoirs, written in French or Latin, should be addressed, free of carriage, before the 1st of October, 1820, to M. Giraudy, Perpetual Secretary of the Society, Rue Traversiere-Saint-Honoré, No. 33, à Paris.

---

The great number of societies which have been formed in different parts of the kingdom within the last thirty or forty years, for the relief of the widows and orphans of medical practitioners, must be a durable, and at the same time very honourable, memorial of the good sense and active benevolence of the members of that profession.

Though the utility of these institutions is indisputable, yet it has, unfortunately, too often happened that widows and orphans are not the only persons connected with this profession who have been the subjects of want and distress, but that practitioners themselves have become, from various causes, exposed to so many difficulties and privations, as to have been equally, if not in a superior degree, objects of commiseration.

We have great pleasure, therefore, in announcing to the medical public, that an institution was founded about three years ago, under the name of the *Medical Benevolent Society*, for the purpose of raising a fund for the relief and assistance of such of its members who may, through want of success in business, or unforeseen misfortunes, be so reduced in their circumstances as to stand in need of pecuniary aid. This society is ready to receive into its number regular practitioners in every branch of the profession throughout England and Wales. The subscription of one guinea at admission, and the same annually, paid in advance, constitutes a member, and entitles him, in case of need, to such advantages as the future state of the fund shall be able to afford. It must surely, then, be considered as a duty incumbent upon every medical man, without any exception, to unite with such an institution. The more opulent, for the benefit of that class of their brethren (which is, alas! too numerous,) whose situation is that of entire dependence upon the uncertainty of professional success; and the latter class, as not knowing but that ill health, infirmity, or old age, may overtake them, before they shall have been able to make provision for a period of life which must, from these causes, be utterly unable to provide for itself.

Though this society is but in its infancy, it consists already of between 200 and 300 members, and is under the especial patronage of his Royal Highness the Duke of Sussex. Dr. John Latham, president of the Royal College of Physicians, presides over it; and its vice-presidents are, Dr. Hull, of Manchester; Henry Cline, Esq. surgeon; and Arthur Tegart, Esq. apothecary to their Royal Highnesses the

Dukes of Clarence and Kent. Among its members will be found the names of Drs. Baillie, Ainslie, and Babington; and Messrs. Abernethy and Astley Cooper, surgeons. At the quarterly meeting of the directors held in September, seven new members were ballotted for and admitted.

In addition to the Benevolent Fund above mentioned, there is, under the same board of managers, another fund, totally distinct from the former, for the purpose of granting annuities of £50. or £100. during the lives of medical subscribers to it, after they shall have attained the age of sixty years. The payments for this purpose are regulated by the age of subscribers at the time of their admission, agreeably to well-known rules of calculation, settled under the direction of an eminent mathematician, and may be made either in one sum, or by annual instalments, at the option of the party insuring. Further particulars, relative to both these funds, may be known by application to the Secretary, Mr. H. C. Field, Surgeon, 95, Newgate-street, London.*

### LECTURES ANNOUNCED TO COMMENCE IN OCTOBER.

* This notice is inserted here at the request of the Committee of the Society.—Edit.

### REPORT OF DISEASES.

UNTIL towards the middle part of the past month, when the wind blowing for some days from the north produced a sudden and extensive change in the temperature of the atmosphere, the season had been unusually healthy. Diarrhœa has been rather frequent, though not in general severe; but cholera has not at any time been very prevalent: neither have dysentery nor gastric fever been of common occurrence. Cholera and diarrhœa were said by SYDENHAM to prevail in autumn, as sure as swallows come in summer; and it is the having escaped the general dispersion of those complaints, that has rendered the present autumn hitherto so peculiarly healthy. A few cases of melæna have occurred; all of them, with two exceptions, in persons habitually affected with disorder of the digestive system. Typhous fever has appeared occasionally, but it has ceased to be so prevalent as to merit the appellation of an epidemic at the present time. Autumnal gastric and intestinal disorders are commonly attributed to the extensive use of fruit as aliment; but the same causes which contribute to render autumnal fruit plentiful, are more probably those which give rise to the disorders to which we allude,—great and long-continued heat and dryness of the atmosphere. This is expressly the case with cholera, the origin of which seems to depend chiefly on affection of the liver sympathetic with the state of the skin. Here we probably witness effects that might be frequently prevented by the habitual use of baths; but we seem to have too much of the disposition dependant on our northern origin left amongst us, to let us yet enjoy this salubrious luxury in a familiar manner. The treatment we have observed to be most generally successful in cholera, has been a mixture of an ounce of sulphate of magnesia with one or two grains of tartar emetic, taken at divided doses in the first twenty-four hours, with the free use of cool diluent drinks. In two instances, where it could be employed conveniently, the use of a bath of from 105° to 110° (Fahrenheit), repeated several times, was employed with prompt and decisive benefit.* Small doses of tartar emetic, with opium and rhubarb, have constituted the remedies subsequently employed.

On the change of the weather to which we have alluded, pulmonic inflammation became rather frequent; and we have seen several old persons, whose lungs were previously diseased, fall victims to it. In one of these cases, affecting a female about 60, it was accompanied with inflamed throat, rapidly running into sloughing ulceration. This is a combination of disease that seems not to have been often witnessed of late years. An extreme state of asperity of the tongue, and small weak pulse, marked the case from the onset. It was also attended with panting respiration, without much pain in the chest, and but occasional fits of coughing. Bleeding was freely resorted to in the first three days, and the blood was cupped and sizy; but it did not produce the slightest temporary alleviation of the disease.

* We were led to the use of this remedy by the recommendation of Colonel FULLARTON, who stated to us that he had seen a hot-bath of much higher temperature than that above designated frequently used in cholera, at Trinidad, with extraordinary advantage. It was there employed so as nearly to produce inflammation of the skin, and the patient kept in it as long and as frequently as could be done without inducing fainting: this, however, was not often produced.

### METEOROLOGICAL JOURNAL.

By Messrs. WILLIAM HARRIS and Co. 50, Holborn, London.

*From August 20 to September 19, 1819, inclusive.*

| Day of Month. | Moon. | Rain guage | THERM. | | | BAROM. | | De Luc's HYGROM. Dry | Damp | | WIND. | | ATMOSPHERIC VARIATION. | | |
|---|---|---|---|---|---|---|---|---|---|---|---|---|---|---|---|
| Aug. | | | | | | | | | | | | | | | |
| 20 | ● | | 65 | 76 | 61 | 30·20 | 30·20 | | 9 | 10 | NE | NE | Cloud. | Fine | |
| 21 | | | 65 | 72 | 62 | 30·16 | 30·14 | | 10 | 8 | NE | NE | Cloud. | Fine | |
| 22 | | | 67 | 73 | 62 | 30·15 | 30·12 | | 9 | 10 | NE | ENE | Cloud. | Fine | |
| 23 | | | 68 | 76 | 62 | 30·10 | 30·10 | | 15 | 5 | NE | NNE | Fine | | |
| 24 | | | 67 | 78 | 61 | 30·07 | 30·00 | | 9 | 3 | NNE | SE | Fine | | |
| 25 | | | 65 | 77 | 62 | 29·97 | 30·04 | | 8 | 5 | NNE | NE | Fine | | |
| 26 | | | 67 | 76 | 60 | 30·07 | 30·10 | | 8 | 5 | NE | NE | Fine | | |
| 27 | | | 67 | 74 | 62 | 30·06 | 30·03 | | 6 | 8 | NE | NE | Fine | | |
| 28 | ☾ | | 67 | 72 | 62 | 30·00 | 29·83 | | 8 | 8 | ENE | SW | Fine | Cloud. | Fine |
| 29 | | | 69 | 72 | 61 | 29·72 | 29·69 | | 9 | 6 | WSW | WSW | Fine | | |
| 30 | | ,08 | 67 | 70 | 55 | 29·46 | 29·26 | | 9 | 7 | S | W | Fine | Rain | Cloud. |
| 31 | | | 61 | 64 | 49 | 29·40 | 29·45 | | 5 | 5 | W | WSW | Fine | Cloud. | Fine |
| Sep. | | | | | | | | | | | | | | | |
| 1 | | | 53 | 58 | 50 | 29·58 | 29·70 | | 5 | 3 | NW | W | Fine | | |
| 2 | | | 54 | 63 | 50 | 29·75 | 29·75 | | 7 | 8 | W. | WSW | Fine | | |
| 3 | | ,07 | 65 | 69 | 56 | 29·69 | 29·84 | | 12 | 11 | W | WSW | Cloud. | Fine | Rain |
| 4 | ○ | ,05 | 60 | 70 | 62 | 29·98 | 29·93 | | 11 | 10 | W | WSW | Cloud. | Fine | Rain |
| 5 | | ,29 | 62 | 70 | 53 | 29·81 | 29·93 | | 13 | 9 | WNW | W | Rain | Rain | Fine |
| 6 | | | 67 | 65 | 51 | 29·99 | 30·09 | | 9 | 10 | W | WNW | Fine | Fine | Fine |
| 7 | | | 64 | 68 | 64 | 30·08 | 30·10 | | 13 | 14 | W | W | Fine | Fine | Cloud. |
| 8 | | | 68 | 75 | 63 | 30·13 | 30·16 | | 13 | 10 | W | WNW | Fine | | |
| 9 | | | 67 | 71 | 61 | 30·16 | 30·13 | | 12 | 11 | SSW | WSW | Cloud. | Fine | |
| 10 | | | 65 | 70 | 55 | 30·12 | 30·07 | | 12 | 8 | ESE | SE | Fine | | |
| 11 | ☽ | | 60 | 66 | 58 | 30·12 | 30·18 | | 10 | 11 | E | NE | Cloud. | Fine | |
| 12 | | | 63 | 68 | 51 | 30·25 | 30·30 | | 9 | 6 | ENE | ENE | Fine | | |
| 13 | | | 58 | 66 | 54 | 30·30 | 30·28 | | 8 | 8 | NE | SE | Fine | | |
| 14 | | | 59 | 64 | 57 | 30·25 | 30·17 | | 10 | 9 | ESE | E | Foggy | Fine | Fine |
| 15 | | ,06 | 60 | 72 | 50 | 30·00 | 29·87 | | 11 | 12 | E | SSW | Foggy | Cloud. | Rain |
| 16 | | ,19 | 50 | 55 | 47 | 29·75 | 29·35 | | 10 | 7 | NNW | N | Cloud. | Rain | Fine |
| 17 | | | 52 | 60 | 55 | 30·00 | 30·15 | | 9 | 7 | NNE | N | Fine | Fine | Fine |
| 18 | | | 60 | 64 | 53 | 30·23 | 30·20 | | 10 | 10 | NNW | N | Foggy | Fine | Fine |
| 19 | ● | | 59 | 64 | 46 | 30·20 | 30·26 | | 7 | 5 | NE | NE | Fine | Fine | Fine |

The quantity of rain fallen in August, is 34-100ths of an inch.

